# SerotoninAI: Serotonergic System Focused, Artificial Intelligence-Based Application for Drug Discovery

**DOI:** 10.1021/acs.jcim.3c01517

**Published:** 2024-01-30

**Authors:** Natalia Łapińska, Adam Pacławski, Jakub Szlęk, Aleksander Mendyk

**Affiliations:** † Department of Pharmaceutical Technology and Biopharmaceutics, Jagiellonian University Medical College, 30-688 Kraków, Poland; ‡ Doctoral School of Medicinal and Health Sciences, Jagiellonian University Medical College, 30-688 Kraków, Poland

## Abstract

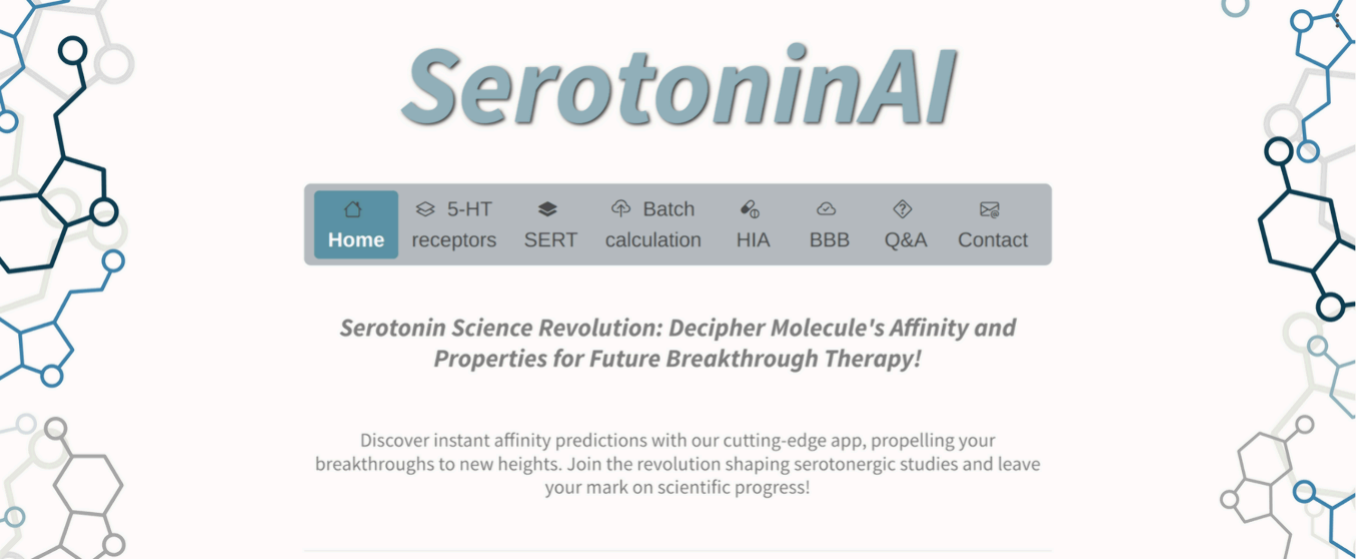

SerotoninAI is an innovative web application for scientific purposes focused on the serotonergic system. By leveraging SerotoninAI, researchers can assess the affinity (pKi value) of a molecule to all main serotonin receptors and serotonin transporters based on molecule structure introduced as SMILES. Additionally, the application provides essential insights into critical attributes of potential drugs such as blood–brain barrier penetration and human intestinal absorption. The complexity of the serotonergic system demands advanced tools for accurate predictions, which is a fundamental requirement in drug development. SerotoninAI addresses this need by providing an intuitive user interface that generates predictions of pKi values for the main serotonergic targets. The application is freely available on the Internet at https://serotoninai.streamlit.app/, implemented in Streamlit with all major web browsers supported. Currently, to the best of our knowledge, there is no tool that allows users to access affinity predictions for serotonergic targets without registration or financial obligations. SerotoninAI significantly increases the scope of drug development activities worldwide. The source code of the application is available at https://github.com/nczub/SerotoninAI_streamlit.

## 1 Introduction

The rise and development of artificial intelligence (AI) tools have a revolutionary impact on many areas of science and industry. In the field of drug design, AI is showing the potential to change traditional approaches, enabling faster and more efficient discovery and development of new therapeutic agents.^1,2^ One of the benefits of AI is the development of advanced QSAR (quantitative structure–activity relationship) and QSPR (quantitative structure–property relationship) models. Their ability to screen new active compounds, possible side effects, bioavailability and other properties can speed up and increase cost effectiveness both of the drug discovery and development phases.^3−6^

Our work focuses on serotonin receptors and transporters. Selected biological targets are important for many drugs that impact the central nervous system (CNS). Substances that affect the serotonergic system are used to treat mood, migraine, regulate sleep, and treat neurotic disorders or eating disorders. Furthermore, recent studies show that they can improve cognitive function. Preliminary studies also show that they may represent an even broader spectrum of therapeutic applications than CNS activity. For example, the serotonin 5-HT2C receptor has a role in impulse control, and the 5-HT3 receptor plays a role in controlling nausea and vomiting.^7−9^

SerotoninAI is an application focused on compounds with a serotonergic activity. It allows predictions of affinity for various 5-HT receptors as well as the serotonin transporter. The affinity for a selected biological target ensures a potential application of a molecule as a future drug and allows us to anticipate possible side effects. Moreover, SerotoninAI predicts human intestinal absorption (HIA) and blood–brain barrier (BBB) penetration of the given chemical structure. For the molecule, a confirmed BBB penetration is a crucial property for drugs acting on the central nervous system, whereas extensive HIA ensures feasibility of the oral administration route, the most preferred and acceptable one in the therapy. The artificial intelligence (AI)-based system for HIA has been developed on data sets with serotonergic ligands, which limits its application to these molecules only.^10^

There are a number of ready-to-use programs for assessing protein–molecule interactions which the main task is to dock molecules in the corresponding protein, such as NovaDock^11^ and AutoDock.^12^ On the other hand, VEGA QSAR^13^ and QSAR TOOLBOX^14^ do not provide predictions of affinity for selected receptors. Their application focuses on physical and chemical properties and also ADME-TOX properties. In contrast, typical QSAR models are provided by applications such as QSAR-Co^15^ and PharmQSAR.^16^ In the case of QSAR-Co, the prediction capabilities are limited to classification models. Moreover, to obtain predictions, the user needs to download and run the tool’s own machine, which might introduce some difficulties related to OS/machine specification. Moreover, the authors of the application do not provide ready-to-use models related to the serotonin system. PharmQSAR allows the creation of statistical models using techniques CoMFA (Comparative Molecular Field Analysis) and CoMSIA (Comparative Molecular Similarity Indices Analysis)^17^ and also does not provide models for serotonin receptors and transporters. In the aforementioned applications for QSAR models, whether ready-made or with the ability to create them, registration is required, which greatly limits the use of such an application. Provided preliminary limitations for users prevent us from directly comparing the presented systems with our models.

The only tool available without registration that provides QSAR models is aiQSAR.^18^ In order to use the ready-made models, we need to download the applications to our own machine. However, among the 38 biological targets, there is none related to the serotonin system. A publication describing QSAR models for serotonin receptors is the work of Kausar et al.^19^ They describe the results for most serotonin receptors, but we have no way of comparing the results because databases are not available (no information about training and test set beyond the number of molecules). When considering the available tools, we decided to create SerotoninAI as the missing piece to accelerate the drug discovery process.

SerotoninAI as a web-based app allows users to easily obtain predictions for chosen molecules without the need for installing any tools on their own computers, as well as without any programming skills. To the best of our knowledge, SerotoninAI is the first affinity prediction tool for serotonin targets without mandatory registration of a user. Moreover, the only information needed from the user side is SMILES (Simplified Molecular Input Line Entry Specification) of a molecule. It is worth mentioning that the application is distributed under the GNU General Public License version 3^20^ and the source code is provided on the GitHub platform (https://github.com/nczub/SerotoninAI_streamlit).

## 2 Results and Discussion

### 2.1 Databases

The application uses QSAR models developed on data extracted from two leading databases, namely, ZINC and ChEMBL (data extracted in December 2022).^21,22^ Data were acquired for each target involving serotonergic activity, that is, 5-HT1A, 5-HT1B, 5-HT1D, 5-HT2A, 5-HT2B, 5-HT2C, 5-HT3, 5-HT4, 5-HT5A, 5-HT6, 5-HT7, and the serotonin transporter SERT. Despite the presence of ligands for 5-HT1E and 5-HT1F as well as 5-HT5B receptors these databases were not used to create models due to their small size (<500 molecules) resulting in the limited applicability domain. Our approach was to provide models based only on the experimentally validated data; therefore, no decoys were used in any of the training or testing data sets.^23^

**Figure 1:**
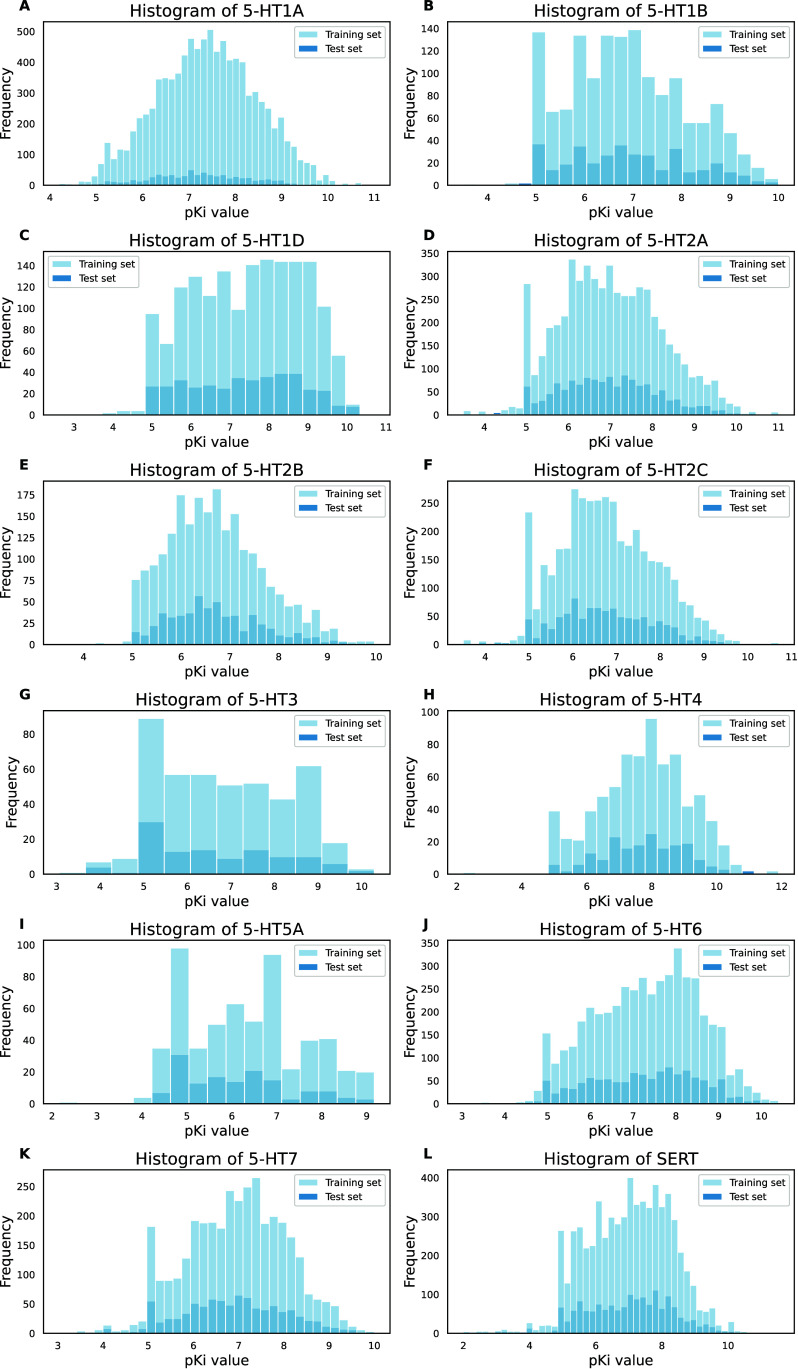
Histograms of the pKi values: (A) 5-HT1A serotonin receptor. (B) 5-HT1B serotonin receptor. (C) 5-HT1D serotonin receptor. (D) 5-HT2A serotonin receptor. (E) 5-HT2B serotonin receptor. (F) 5-HT2C serotonin receptor. (G) 5-HT3 serotonin receptor. (H) 5-HT4 serotonin receptor. (I) 5-HT5A serotonin receptor. (J) 5-HT6 serotonin receptor. (K) 5-HT7 serotonin receptor. (L) Serotonin transporter SERT.

The dependent variable was the value of ligand affinity presented as the negative logarithm of the inhibition constant, the pKi value. In the data preparation part, duplicates were first removed from the ZINC and ChEMBL databases separately, and invalid SMILES were removed. No other specific structure curation was employed. The two databases were then merged, and some molecules were present in both databases. Molecule pairs with a pKi difference greater than 0.1 were removed from the database. In the group of molecule pairs with a pKi difference equal to or less than 0.1, the ZINC molecule was selected as the reference substance. The databases were randomly divided into training and test sets at a ratio of 80:20. The former part of the data (80%) was used to develop QSAR models according to the 10-fold cross validation scheme, and the latter (20%) was used as an external test set. Figure 1 shows histograms of pKi values for each biological target according to the training and test set.

The database for predicting HIA was described in our previous work;^10^ the source of the data was literature data and the ChEMBL database.

QSPR classification model of BBB permeability was based on the database collected from the work of Miao et al.^24^ (data set 1 and data set 2) and enriched by us with data from OCHEM database^25^ (data extracted in July 2023). It resulted in 2853 molecules in the training set. An independent test set from the article by Miao et al. remained in the test set of our experiment (161 molecules) in order to better compare our developed QSPR model.

All data are available in the Supporting Information as a single database.zip file containing separate training/test csv files for each respective biological target.

### 2.2 Molecular Representation

Mordred descriptors were used as a molecular representation of chemical structures.^26^ Mordred effectiveness in this field and suitability for interpretation has been tested in our previous work.^10^ QSAR and QSPR models were created based on two-dimensional descriptors to prevent the risk of variability in three-dimensional descriptors due to nonconvergent molecules’ optimization. Based on SMILES, SerotoninAI automatically creates the molecular descriptors required by the models.

### 2.3 QSAR and QSPR Models

Modeling was performed with the mljar-supervised tool, which belongs to the field of Automated Machine Learning (AutoML).^27^ In the case of receptors, serotonin transporter and BBB permeability models were de novo created for the purpose of the presented application. On the other hand, for human intestinal absorption, the AI-based system has already been published^10^ and has been implemented in this application without any further modifications.

QSAR regression models were created according to the 10-fold cross-validation scheme (10-CV) on 80% of the curated databases. For each target, about 2000 models were developed. We used root-mean-square error (RMSE) and coefficient of determination (*R*^2^) values to evaluate and select the best models (eqs 1 and 2), in accordance with the guidelines of the Organization for Economic Co-operation and Development’s QSAR models.^28^ The results were analyzed for the training set (10-CV) and the separate test set (20%). In Table 1, we show the detailed results for each QSAR model.
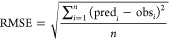
1where RMSE = root-mean-square error, obs_*i*_ and pred_*i*_ = observed and predicted values, *i* = data record number, and *n* = total number of records.
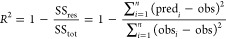
2where *R*^2^ = the coefficient of determination, SS_res_ = the sum of squares of the residual errors, S*S*_tot_ = the total sum of the errors, obs_*i*_ and pred_*i*_ = observed and predicted values, and obs = arithmetical mean of observed values.

**Table 1:** Number of Ligands for Each Target, Number of Descriptors Used for QSAR Models after Removing Constant and Highly Correlated Variables, Results for Training Set and Test Set, and Type of Each Model of QSAR Models

Target	Number of ligands	Number of descriptors	RMSE 10-CV	R^2^ 10-CV	RMSE test	R^2^ test	Model type
5-HT1A	10,175	704	0.555	0.733	0.629	0.660	LightGBM
5-HT1B	1833	710	0.627	0.731	0.558	0.805	Ensemble model (5x Xgboost, 4x LightGBM)
5-HT1D	2072	739	0.566	0.836	0.536	0.858	Ensemble model (5x Xgboost, 4x LightGBM, 2x CatBoost)
5-HT2A	6926	703	0.582	0.769	0.567	0.764	Xgboost
5-HT2B	2821	723	0.561	0.674	0.607	0.608	Ensemble model (6x Xgboost, 2x LightGBM, 5x CatBoost)
5-HT2C	5097	720	0.625	0.673	0.603	0.685	LightGBM
5-HT3	562	665	0.837	0.672	0.867	0.680	Ensemble model (2x Xgboost, 3x CatBoost, 1x LightGBM)
5-HT4	949	516	0.682	0.753	0.690	0.697	Xgboost
5-HT5A	720	704	0.575	0.797	0.568	0.761	Ensemble model (3x Xgboost, 3x CatBoost, 1x ExtraTrees, 1x LightGBM)
5-HT6	5925	688	0.579	0.777	0.624	0.749	Xgboost
5-HT7	4411	727	0.624	0.684	0.601	0.728	CatBoost
SERT	8091	541	0.490	0.851	0.492	0.858	LightGBM

In Table 2, we have presented examples of drugs available on the market along with their affinities from literature sources and pKi values predicted by the SerotoninAI.

**Table 2:** SerotoninAI’s Predictions and Literature Values of pKi for Drug Examples Related to Serotonergic System

Drug	Target	Experimental/Literature pKi value	SerotoninAI prediction
Fluoxetine^29^	5-HT2A	6.150	6.631
	5-HT2C	7.371	6.308
	SERT	8.708	8.013
Aripiprazole^30^	5-HT1A	8.252	7.527
	5-HT1B	6.081	5.913
	5-HT1D	7.167	6.753
	5-HT2A	8.060	8.366
	5-HT2B	9.444	7.982
	5-HT2C	7.119	7.195
	5-HT3	6.202	6.160
	5-HT5A	5.907	5.890
	5-HT6	6.244	6.716
	5-HT7	7.987	7.570
	SERT	5.967	6.646
Zolmitriptan^31^	5-HT1A	7.105	6.562
	5-HT1B	8.377	8.089
	5-HT1D	9.199	8.733
Clozapine^32^	5-HT1A	6.990	6.357

The examples presented above are also included in curated databases prepared to create QSAR models, which do not constitute the usefulness of such a model for unseen examples of molecules. Therefore, we decided to test the created models on the latest available data. For this purpose, we reviewed the literature from 2022, when the first databases were created. New experimental data were available for seven biological targets (5-HT1A, 5-HT1B, 5-HT2A, 5-HT2B, 5-HT2C, 5-HT5A, 5-HT6, and 5-HT7).^33−38^ In Table 3, we present summary results for each receptor and the sum of predictions, using SerotoninAI, located in four categories: when the prediction difference was less than 0.5, greater than or equal to 0.5 and less than 1.0, the third group being prediction differences equal to or greater than 1.0 and less than 2.0, and the last group indicating a very large prediction difference, a value equal to or greater than 2.0. All predictions are available in Supporting Information S1. The worst predictions are seen for the 5-HT5A receptor. In our opinion, this is due to too little differentiated data. Of the seven targets for which external data were available, the database for the 5-HT5A receptor was the smallest (720 molecules).

**Table 3:** Summary of pKi Pedictions by SerotoninAI for New Experimental Data Available in the Literature in the Years 2022–2023

	Differences of literature data and SerotoninAI predictions of pKi			
receptor	<0.5	≥0.5 and <1.0	≥1.0 and <2.0	≥2.0
5-HT1A^38^	5	2	13	1
5-HT1B^33^	1	–	–	–
5-HT2A^33,34,36,37^	31	15	6	–
5-HT2B^33^	3	1	–	–
5-HT2C^34^	10	4	–	–
5-HT5A^35^	5	–	1	6
5-HT6^33,36,37^	24	11	8	1
5-HT7^33,36,37^	23	11	10	–
total	102	44	38	8

**Figure 2:**
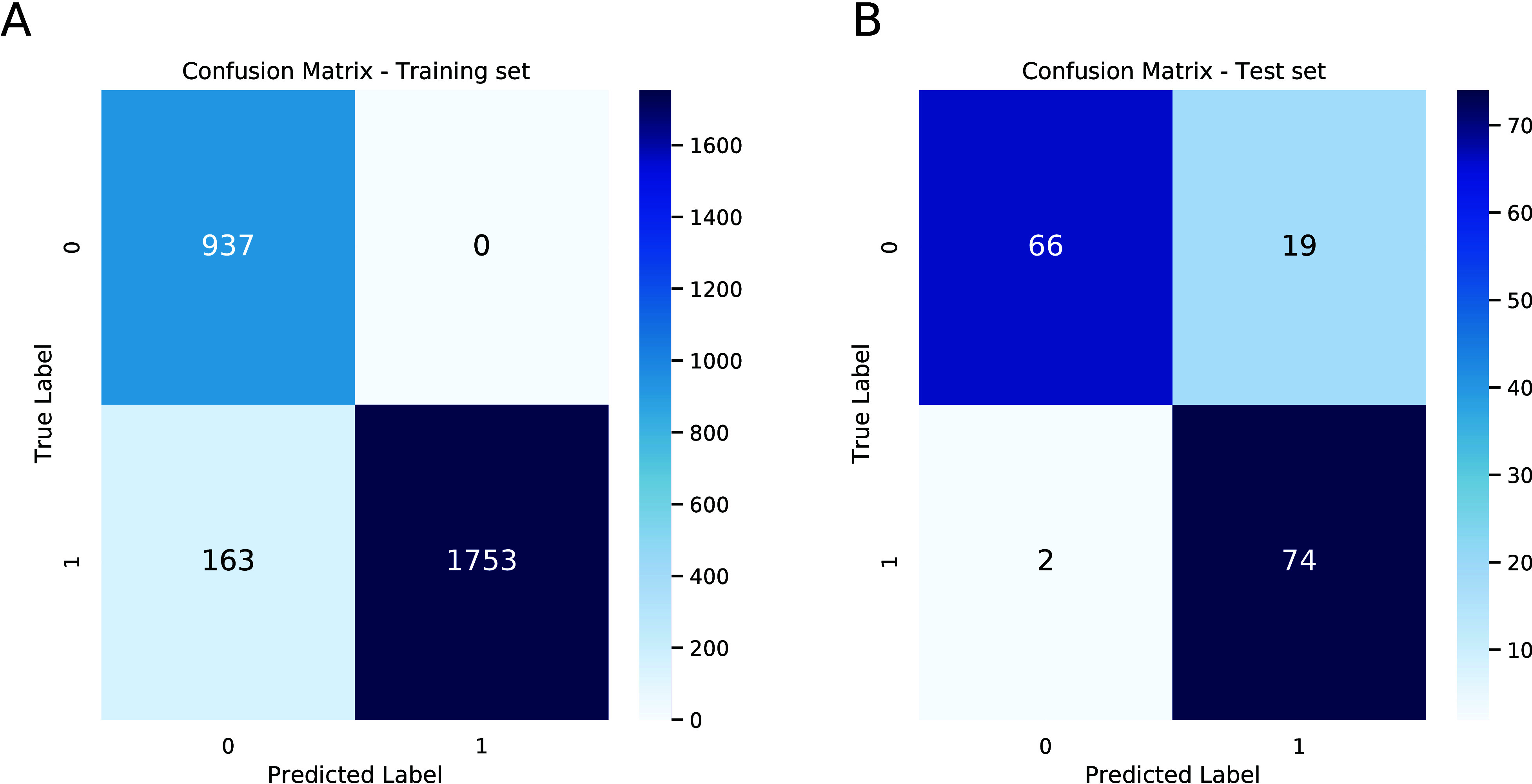
Confusion matrices for training and test sets: blood–-brain barrier QSPR model.

The QSPR model for BBB penetration was a binary classification model (1, molecule penetrates to brain; 0, no penetration). It was also created according to 10-CV. To evaluate the model, classification metrics like accuracy, precision, recall, F1 score, and Matthews correlation coefficient (MCC) were used (eqs 3–7). These results and confusion matrices for the training and test set are shown in Table 4 and Figure 2. We compared our results on the test set with the recent article on BBB penetration,^24^ and our model resulted in more accurate predictions. Supporting Information S2 provides a detailed description of these results.
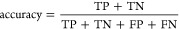
3
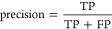
4
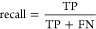
5
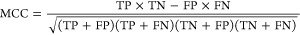
6where TP = true positive, TN = true negative, FP = false positive, and FN = false negative.
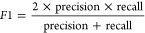
7

**Table 4:** Summary of Classification Metrics for Training and Test Set for the Blood–Brain Barrier Penetration Model

	Training set (10-CV)	Test set
Accuracy	0.94	0.87
Precision	1.00	0.80
Recall	0.91	0.97
F1 score	0.96	0.88
Matthews correlation coefficient	0.88	0.76
Number of compounds	2853	161
Type of model	Ensemble model (5x Xgboost, 7x NeuralNetwork, 2x LightGBM)	

### 2.4 Applicability Domain

The major challenge that researchers face in developing QSAR or QSPR models is the scarcity of data. Based on the available data, the model predicts correctly within the relevant ranges of descriptors. For these reasons, SerotoninAI with each prediction provides information on whether the studied compound is within the applicability domain (AD) or not. Naturally, within-AD predictions are characterized with higher accuracy even though the compound is unknown to the system, namely, beyond the training data.

**Figure 3:**
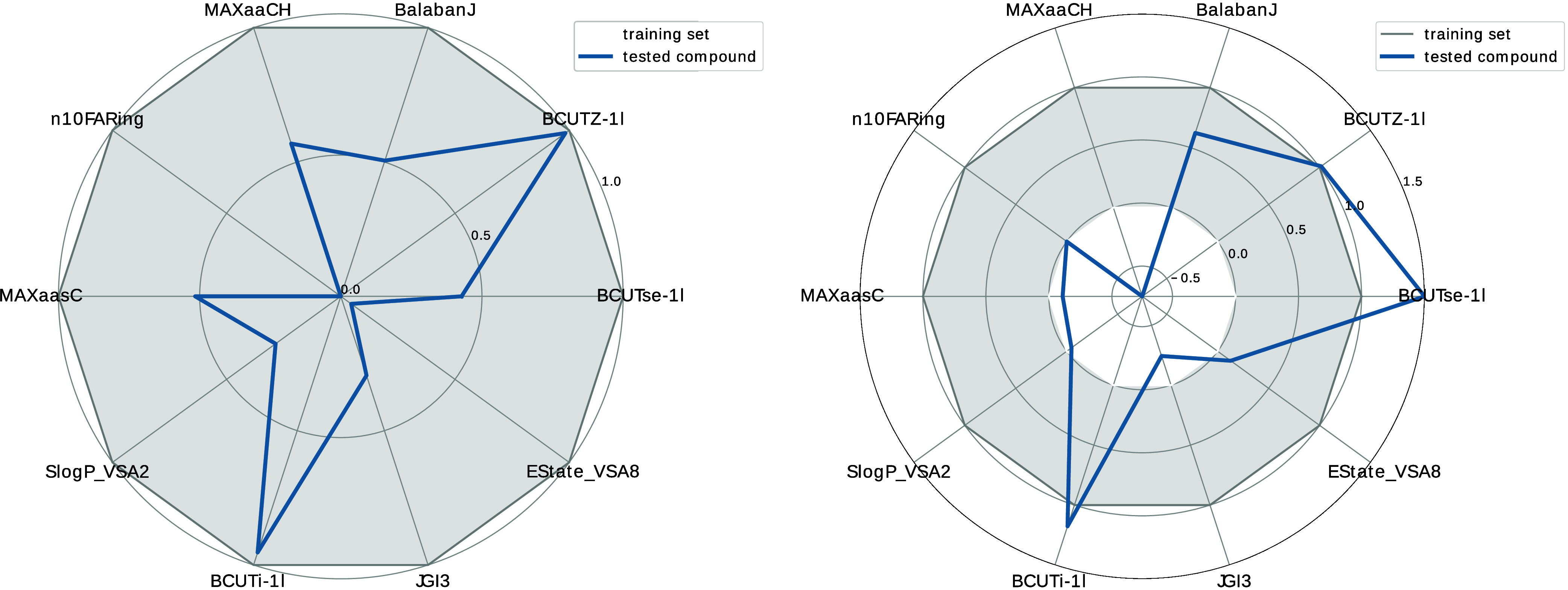
A comparison of the molecule’s applicability domains. (Left) A molecule within the applicability domain. (Right) A molecule out of the applicability domain. The gray area indicates the application domain.

The AD was obtained based on the 10 most important descriptors for each model. The importance ranking of the descriptors was obtained using SHAP analysis. SHAP (Shapley Additive Explanations) is a method used to explain complex AI/ML models. It is based on a concept called the Shapley value, introduced by Lloyd Shapley in 1952.^39^ Shapley, who was distinguished for his input in game theory by the Nobel Memorial Prize in Economic Sciences in 2012, focused on how to fairly divide rewards from the game among cooperating players. The solution for the analyzed problem should allow for splitting the reward of the team among all players in a proportional way to its contribution to the result and at the same time fulfill criteria of efficiency, additivity, symmetry, and null player presence detection. The unique solution for that problem is the Shapley value which divides the reward based on each player’s marginal contribution, ensuring fairness. In the realm of predictive modeling, this framework can be adapted to assess the incremental contribution of each input variable of the model to the predicted outcome.^40^ In this study, we employed SHAP analysis to examine the overarching influence of each variable, considering both the magnitude and the direction (high or low values of the variable) of its impact on the final prediction. This analysis was conducted in Python environment, leveraging a framework developed by Szlęk,^41^ further augmented with a wrapper for the mljar package. Summary plots of SHAP analysis for each obtained model are presented in Supporting Information S3, except for the HIA model, where the entire analysis was described in previous article.^10^ The values of these descriptors in the training set were transformed via Min–Max normalization. A compound is under AD if seven descriptors are in the range between minimum and maximum values of the training set. Figure 3 presents examples of compounds within and outside the applicability domain.

### 2.5 Application Development

SerotoninAI was developed using Streamlit, an open-source Python library that simplifies the process of developing web applications. It enables developers to create interactive and user-friendly web applications using Python scripts.^42^ Our app includes pharmacological descriptions for each biological target and a medical and biopharmaceutical introduction to intestinal permeability and blood–brain barrier based on literature data.^7−9,43^ This enables scientists to better understand the mechanisms of action of these receptors and identify potential targets for new drugs. This knowledge can accelerate the drug discovery process, enabling scientists to design compounds with the desired pharmacological properties. The graphics page was enhanced with images created using Canva software.^44^

## 3 Implementation

SerotoninAI operates in several modes. The user can obtain predictions for the target’s affinity or class of HIA and BBB, either for a single compound or based on a collection of molecules represented by a CSV file, the so-called batch mode. However, due to limited resources provided by the Streamlit server, we encourage users to run batch mode locally on their own machines. From the user’s perspective, the model makes predictions based on SMILES, imported directly from the DrugBank/ChEMBL/ZINC without a specific structure curation. In the case of a CSV file, compounds should be represented in a column named “smiles”. There is also the possibility to draw molecules and obtain particular SMILES.^45^

Another feature of the application is to obtain a prediction for one biological target only, multiple user-selected targets, or all of them at once. Depending on the research needs, scientists may choose which serotonin receptors are relevant. This mode works with only a CSV file. It is worth remembering that with more molecules and more targets under study, the prediction time increases. The result of the predictions for multiple molecules is a downloadable file with ready-made predictions. As in the case of individual predictions for a single target, information about the applicability domain appears. In the case of batch mode, it takes the values “True” or “False”, where “True” means that the tested compound is in the AD defined in section 2.4.

## 4 Conclusion

The developed SerotoninAI application is publicly available AI-based software for obtaining affinity and permeability predictions for the intestinal wall and blood–brain barrier. The use of such predictions for serotonin receptors and transporters can significantly accelerate the time to find new compounds with potential pharmacological activity, mainly in the field of central nervous system diseases. This application, governed by the GNU General Public License version 3,^20^ comes with no liability from the authors for the obtained results. The published version of the app is not password protected; neither is the data privacy ensured. For the latter, the user must download the source code and install the app in the controlled environment.

Limitations due to the small amount of data to create models for several serotonin receptors (5-HT1E, 5-HT1E, and 5-HT5B) will be overcome when more data about ligands for these receptors are published. SerotoninAI will be updated on an ongoing basis for at least 2 years when larger databases become available and/or better predictive models can be obtained. Due to limited resources available to streamlit applications, batch mode computations should be run locally.

## Data Availability

The data underlying this study are available in the published article and its Supporting Information, except the data used for developing HIA model, which are available at https://github.com/nczub/HIA_5-HT.

## Supporting Information Available

Supporting Information S1, Table 1: prediction results of external data of ligands for seven targets of serotonin system (XLSX)
Supporting Information S2, Table 2: prediction results of 161 compounds, test set of blood–brain barrier model (XLSX)
Supporting Information S3, Figure: Summary plot of SHAP analysis (PDF)
Databases (ZIP)

## Abbreviations

10-CV: 10-fold cross-validation
5-HT: 5-hydroxytryptamine, serotonin
5-HT_1A_: serotonin 1A receptor
5-HT_1B_: serotonin 1B receptor
5-HT_1D_: serotonin 1D receptor
5-HT_1E_: serotonin 1E receptor
5-HT_1F_: serotonin 1F receptor
5-HT_2A_: serotonin 2A receptor
5-HT_2B_: serotonin 2B receptor
5-HT_2C_: serotonin 2C receptor
5-HT_3_: serotonin 3 receptor
5-HT_4_: serotonin 4 receptor
5-HT_5A_: serotonin 5A receptor
5-HT_5B_: serotonin 5B receptor
5-HT_6_: serotonin 6 receptor
5-HT_7_: serotonin 7 receptor
AD: Applicability domain
AI: Artificial Intelligence
AutoML: Automated Machine Learning
BBB: blood–brain barrier
CNS: central nervous system
HIA: human intestinal absorption
MCC: Matthews correlation coefficient
QSAR: quantitative structure–activity relationship
QSPR: quantitative structure–property relationship
R^2^: coefficient of determination
RMSE: root-mean-square error
SHAP: SHapley Additive exPlanations
SMILES: simplified molecular-input line-entry system
TN: true negative
TP: true positive;
