# Supplementary material for: SerotoninAI: Serotonergic System Focused, Artificial Intelligence-Based Application for Drug Discovery
Source: J Chem Inf Model. 2024 Jan 30;64(7):2150–7. doi: 10.1021/acs.jcim.3c01517 (PMC11005036; doi:10.1021/acs.jcim.3c01517)
Supplement: Supplementary file 3 — ci3c01517_si_003.pdf [file ci3c01517_si_003.pdf]

Supporting Information S3
summary plots of SHAP analysis

SerotoninAI: serotonergic system focused, artificial intelligence-based application for drug discovery

Natalia Łapińska[1,2], Adam Pacławski[1], Jakub Szlęk[1,*], Aleksander Mendyk[1]

[1]Department of Pharmaceutical Technology and Biopharmaceutics, Jagiellonian University Medical College, 30-688 Kraków, Poland

[2] Jagiellonian University Medical College, Doctoral School of Medicinal and Health Sciences

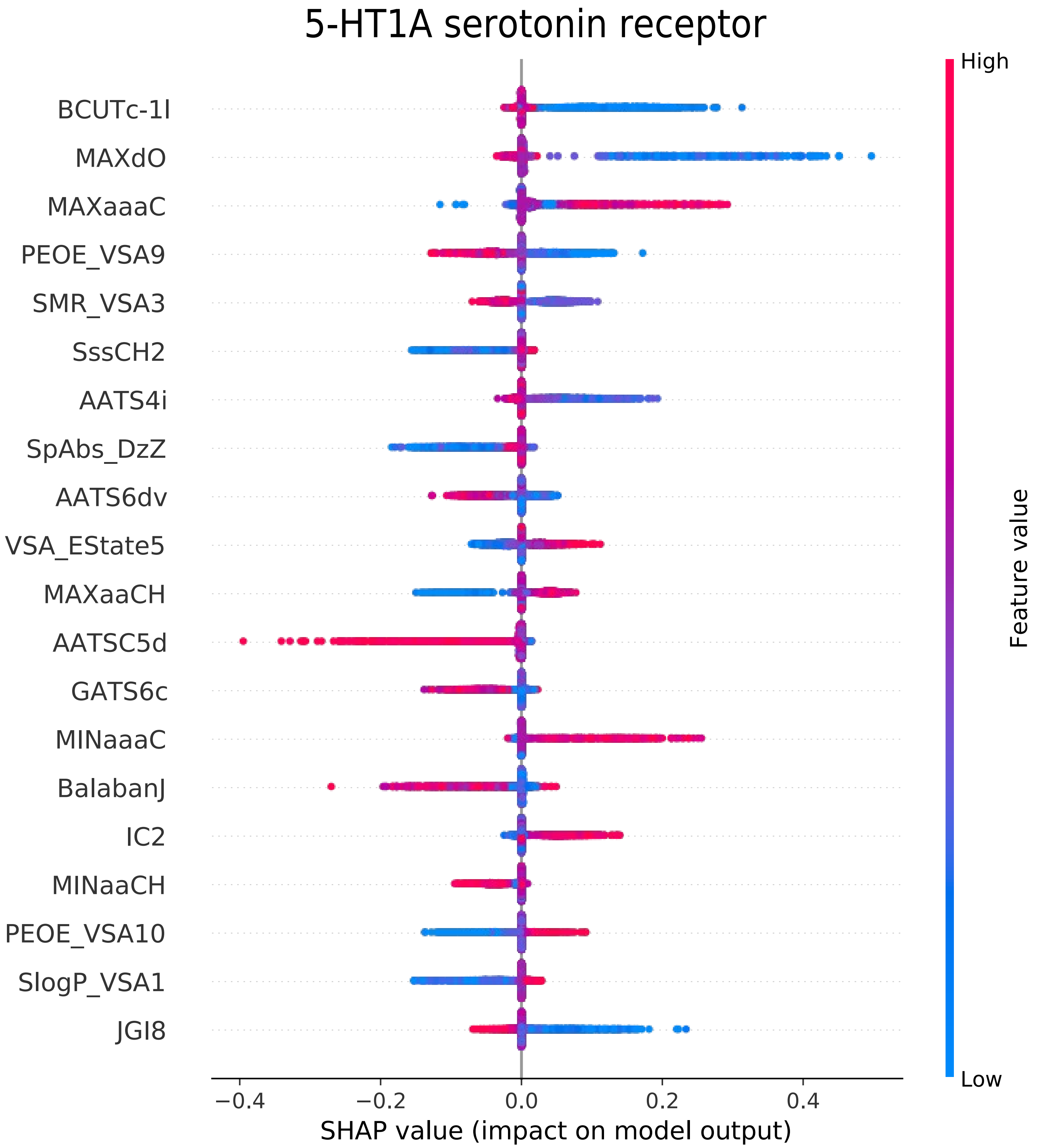

5-HT1A serotonin receptor
BCUTc-1l
MAXdO
MAXaaaC
PEOE_VSA9
SMR_VSA3
SssCH2
AATS4i
SpAbs_DzZ
AATS6dv
VSA_EState5
MAXaaCH
AATSC5d
GATS6c
MINaaaC
BalabanJ
IC2
MINaaCH
PEOE_VSA10
SlogP_VSA1
JGI8
−0.4
−0.2
0.0
0.2
0.4
SHAP value (impact on model output)
High
Low
Feature value

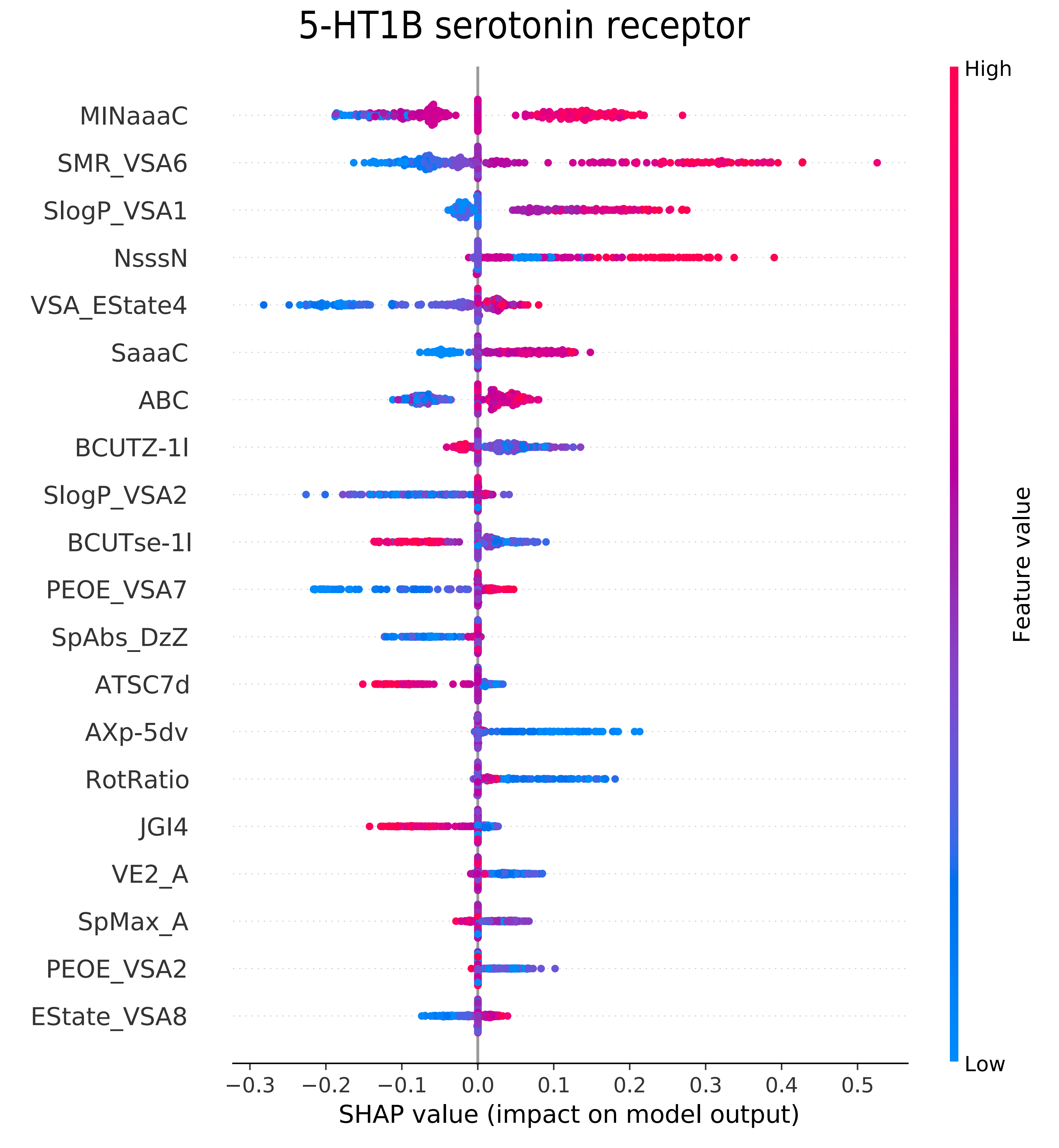

5-HT1B serotonin receptor
MINaaaC
SMR_VSA6
SlogP_VSA1
NsssN
VSA_EState4
SaaaC
ABC
BCUTZ-1l
SlogP_VSA2
BCUTse-1l
PEOE_VSA7
SpAbs_DzZ
ATSC7d
AXp-5dv
RotRatio
JGI4
VE2_A
SpMax_A
PEOE_VSA2
EState_VSA8
−0.3
−0.2
−0.1
0.0
0.1
0.2
0.3
0.4
0.5
SHAP value (impact on model output)
High
Low
Feature value

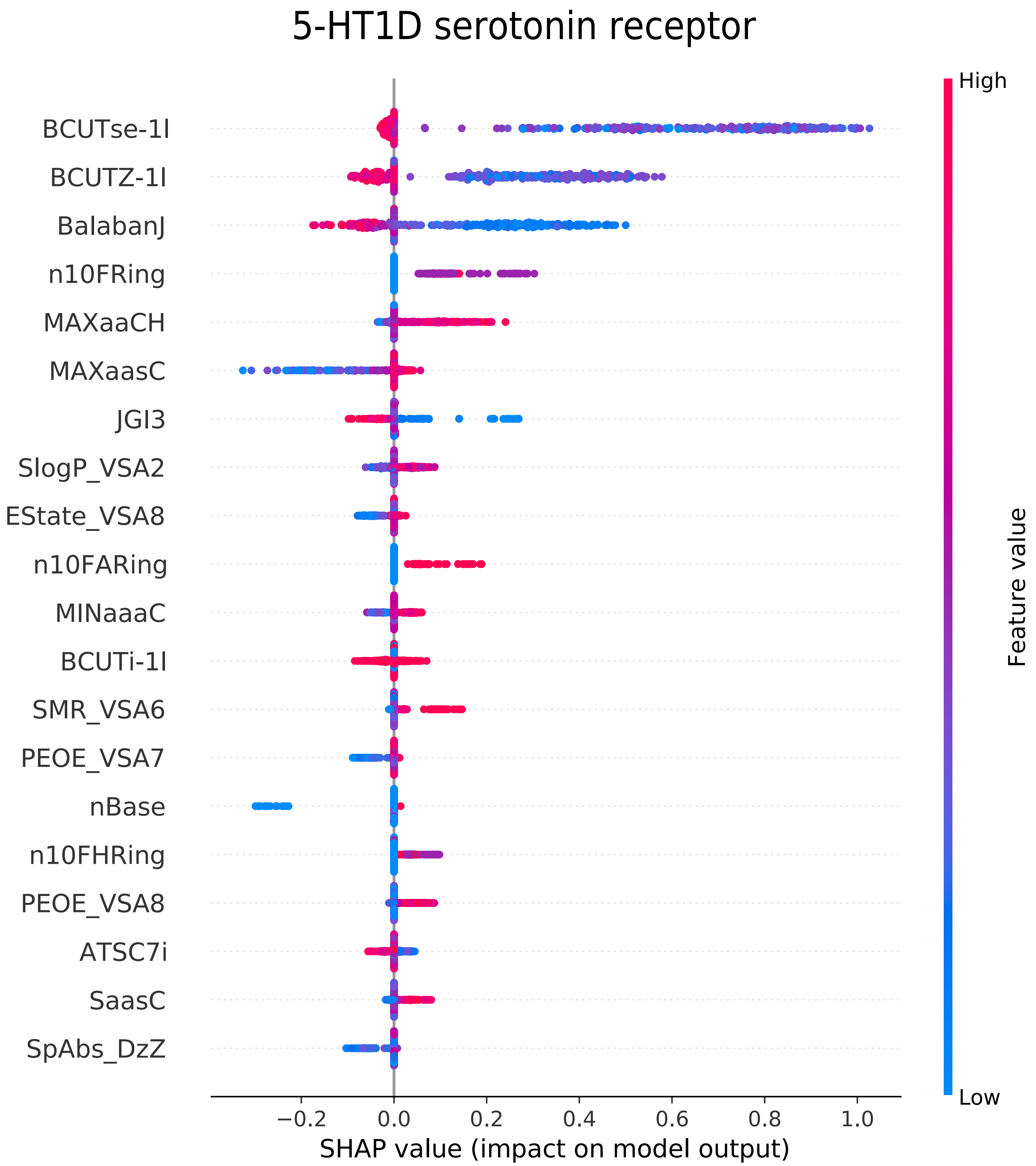
5-HT1D serotonin receptor
BCUTse-1l
BCUTZ-1l
BalabanJ
n10FRing
MAXaaCH
MAXaasC
JGI3
SlogP_VSA2
EState_VSA8
n10FARing
MINaaaC
BCUTi-1l
SMR_VSA6
PEOE_VSA7
nBase
n10FHRing
PEOE_VSA8
ATSC7i
SaasC
SpAbs_DzZ
−0.2
0.0
0.2
0.4
0.6
0.8
1.0
SHAP value (impact on model output)
High
Low
Feature value

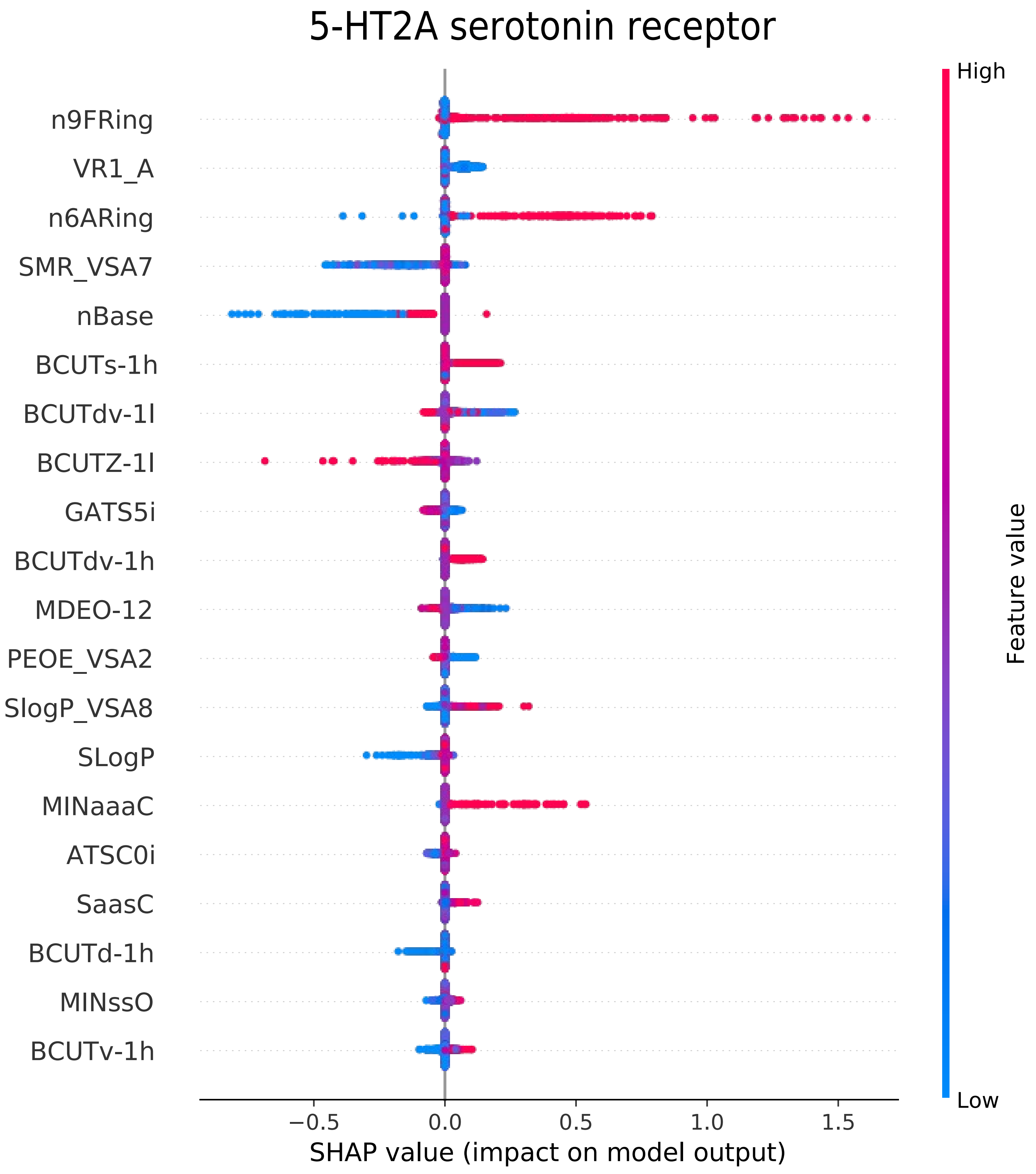
5-HT2A serotonin receptor
n9FRing
VR1_A
n6ARing
SMR_VSA7
nBase
BCUTs-1h
BCUTdv-1l
BCUTZ-1l
GATS5i
BCUTdv-1h
MDEO-12
PEOE_VSA2
SlogP_VSA8
SLogP
MINaaaC
ATSC0i
SaasC
BCUTd-1h
MINssO
BCUTv-1h
−0.5
0.0
0.5
1.0
1.5
SHAP value (impact on model output)
High
Low
Feature value

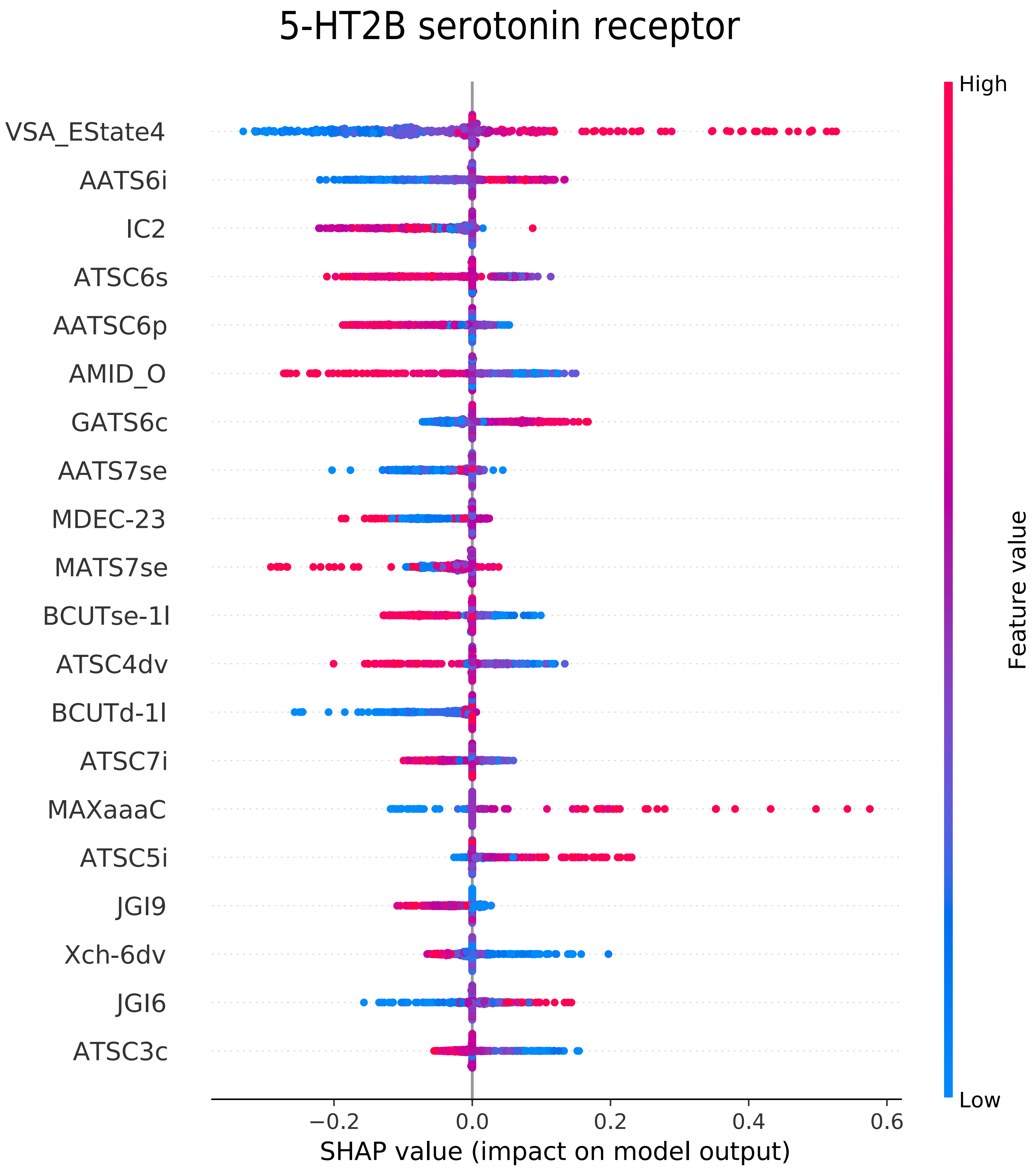

5-HT2B serotonin receptor
VSA_EState4
AATS6i
IC2
ATSC6s
AATSC6p
AMID_O
GATS6c
AATS7se
MDEC-23
MATS7se
BCUTse-1l
ATSC4dv
BCUTd-1l
ATSC7i
MAXaaaC
ATSC5i
JGI9
Xch-6dv
JGI6
ATSC3c
−0.2
0.0
0.2
0.4
0.6
SHAP value (impact on model output)
High
Low
Feature value

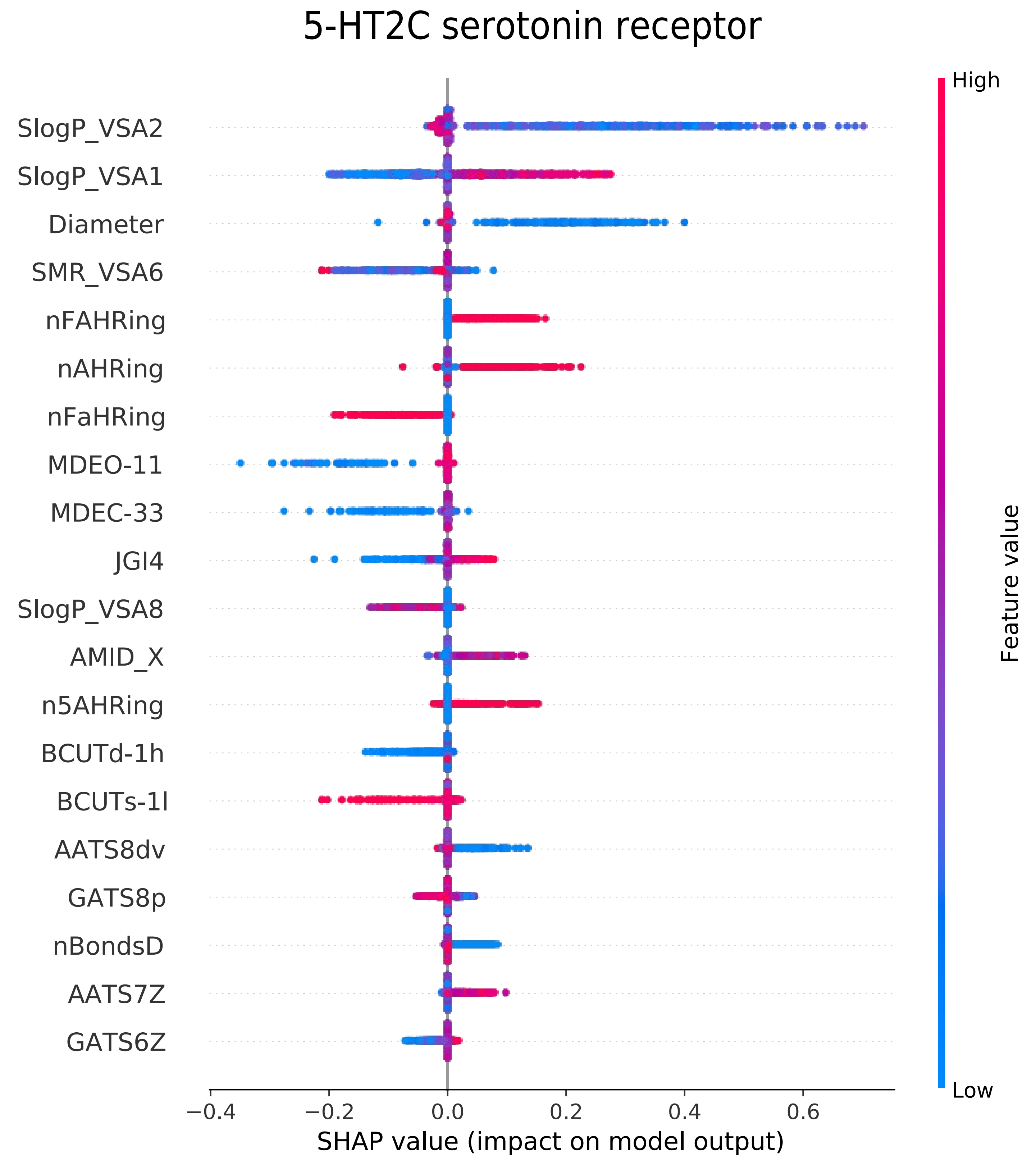
5-HT2C serotonin receptor
SlogP_VSA2
SlogP_VSA1
Diameter
SMR_VSA6
nFAHRing
nAHRing
nFaHRing
MDEO-11
MDEC-33
JGI4
SlogP_VSA8
AMID_X
n5AHRing
BCUTd-1h
BCUTs-1l
AATS8dv
GATS8p
nBondsD
AATS7Z
GATS6Z
−0.4
−0.2
0.0
0.2
0.4
0.6
SHAP value (impact on model output)
High
Low
Feature value

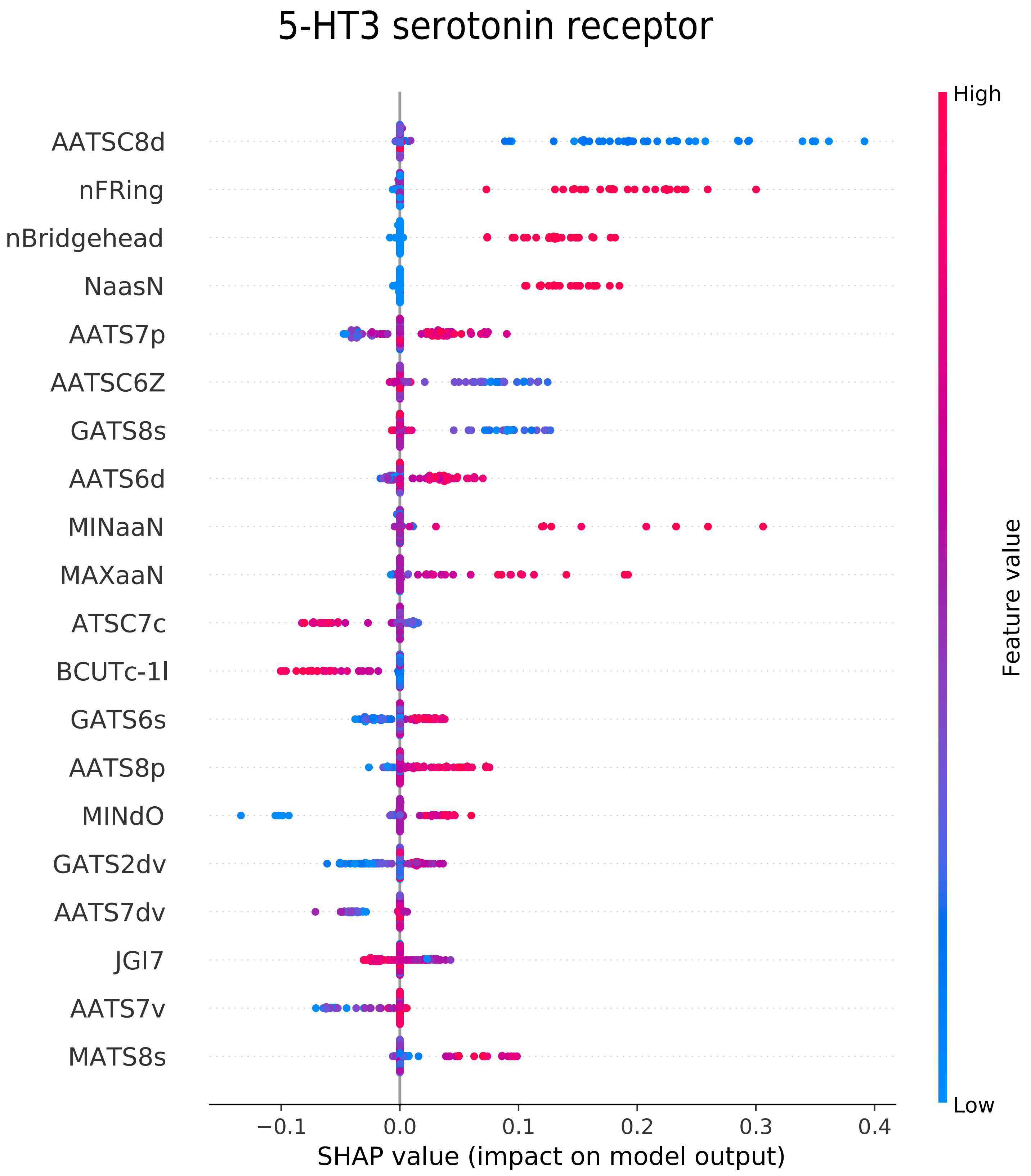
5-HT3 serotonin receptor
AATSC8d
nFRing
nBridgehead
NaasN
AATS7p
AATSC6Z
GATS8s
AATS6d
MINaaN
MAXaaN
ATSC7c
BCUTc-1l
GATS6s
AATS8p
MINdO
GATS2dv
AATS7dv
JGI7
AATS7v
MATS8s
−0.1
0.0
0.1
0.2
0.3
0.4
SHAP value (impact on model output)
High
Low
Feature value

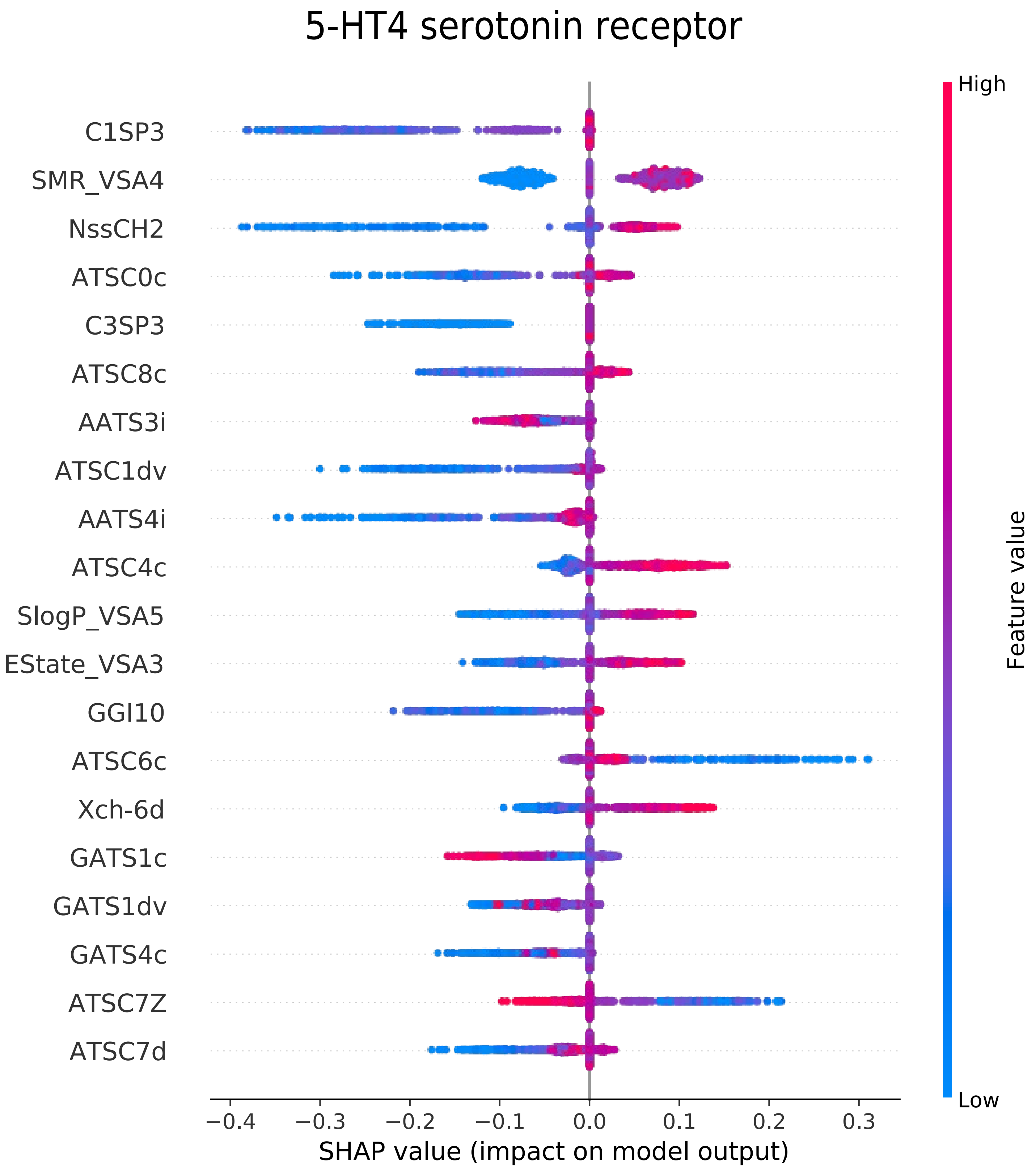
5-HT4 serotonin receptor
C1SP3
SMR_VSA4
NssCH2
ATSC0c
C3SP3
ATSC8c
AATS3i
ATSC1dv
AATS4i
ATSC4c
SlogP_VSA5
EState_VSA3
GGI10
ATSC6c
Xch-6d
GATS1c
GATS1dv
GATS4c
ATSC7Z
ATSC7d
−0.4
−0.3
−0.2
−0.1
0.0
0.1
0.2
0.3
SHAP value (impact on model output)
High
Low
Feature value

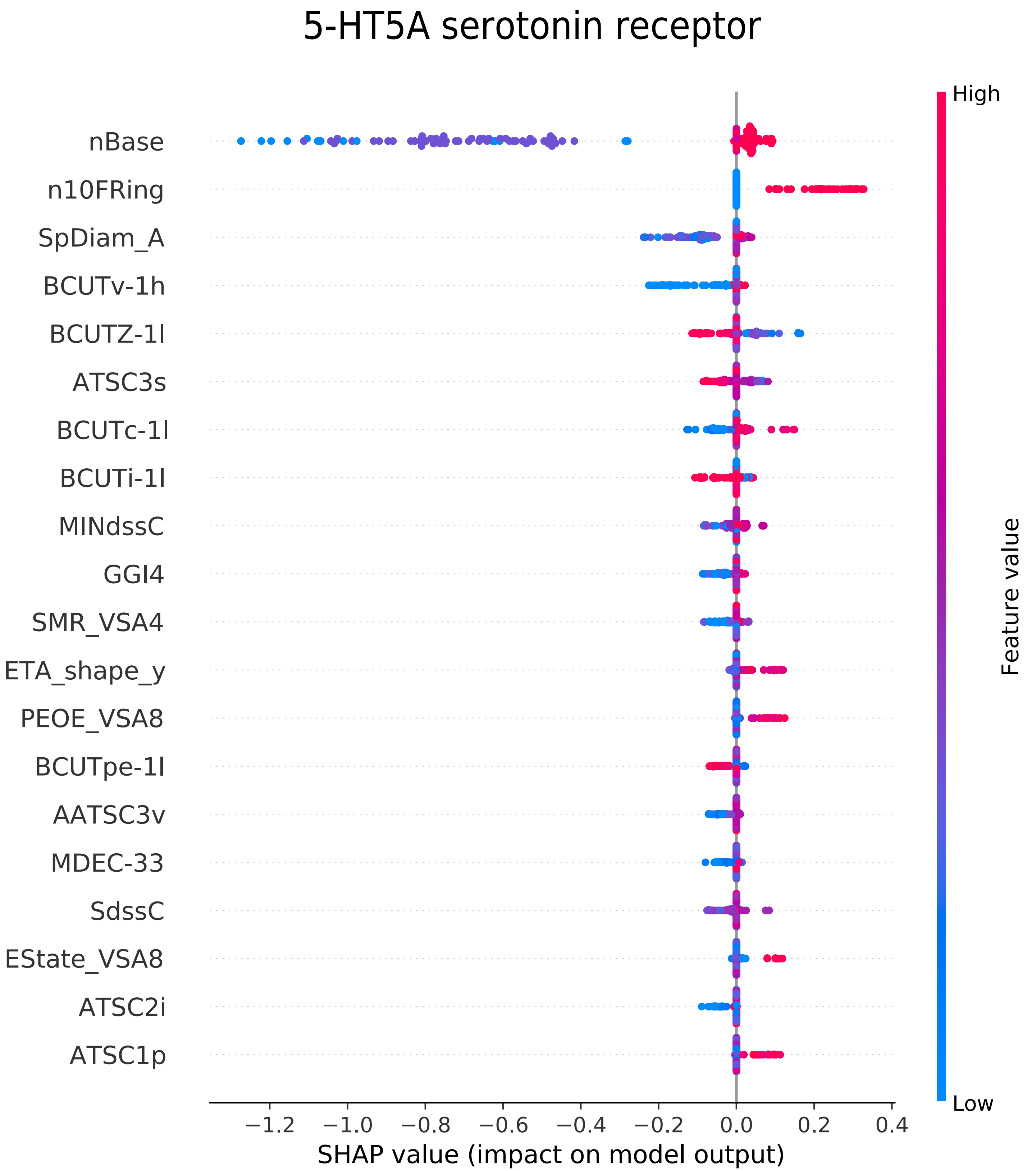
5-HT5A serotonin receptor
nBase
n10FRing
SpDiam_A
BCUTv-1h
BCUTZ-1l
ATSC3s
BCUTc-1l
BCUTi-1l
MINdssC
GGI4
SMR_VSA4
ETA_shape_y
PEOE_VSA8
BCUTpe-1l
AATSC3v
MDEC-33
SdssC
EState_VSA8
ATSC2i
ATSC1p
−1.2 −1.0 −0.8 −0.6 −0.4 −0.2 0.0 0.2 0.4
SHAP value (impact on model output)
High
Low
Feature value

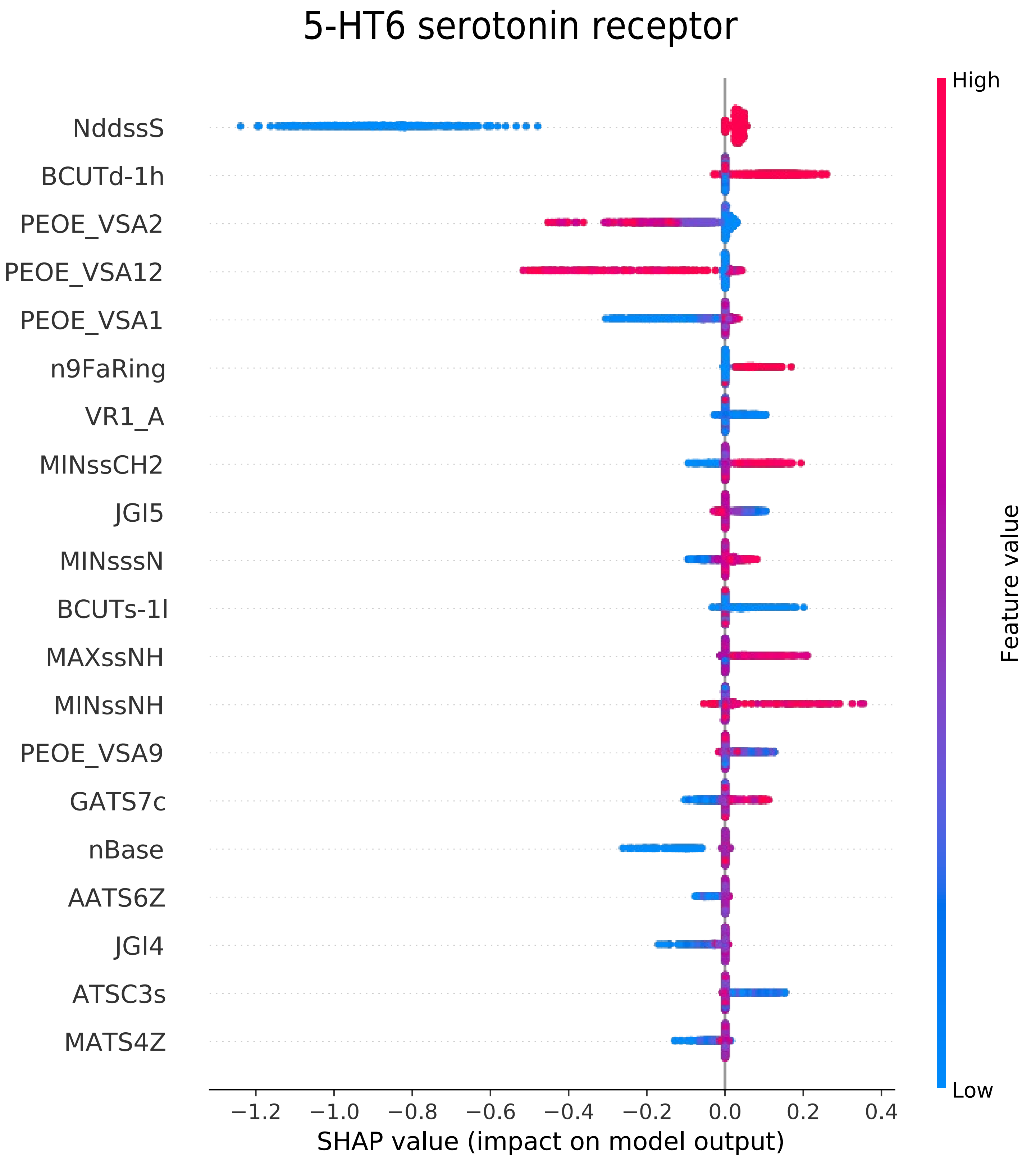
5-HT6 serotonin receptor
NddssS
BCUTd-1h
PEOE_VSA2
PEOE_VSA12
PEOE_VSA1
n9FaRing
VR1_A
MINssCH2
JGI5
MINsssN
BCUTs-1l
MAXssNH
MINssNH
PEOE_VSA9
GATS7c
nBase
AATS6Z
JGI4
ATSC3s
MATS4Z
−1.2
−1.0
−0.8
−0.6
−0.4
−0.2
0.0
0.2
0.4
SHAP value (impact on model output)
High
Low
Feature value

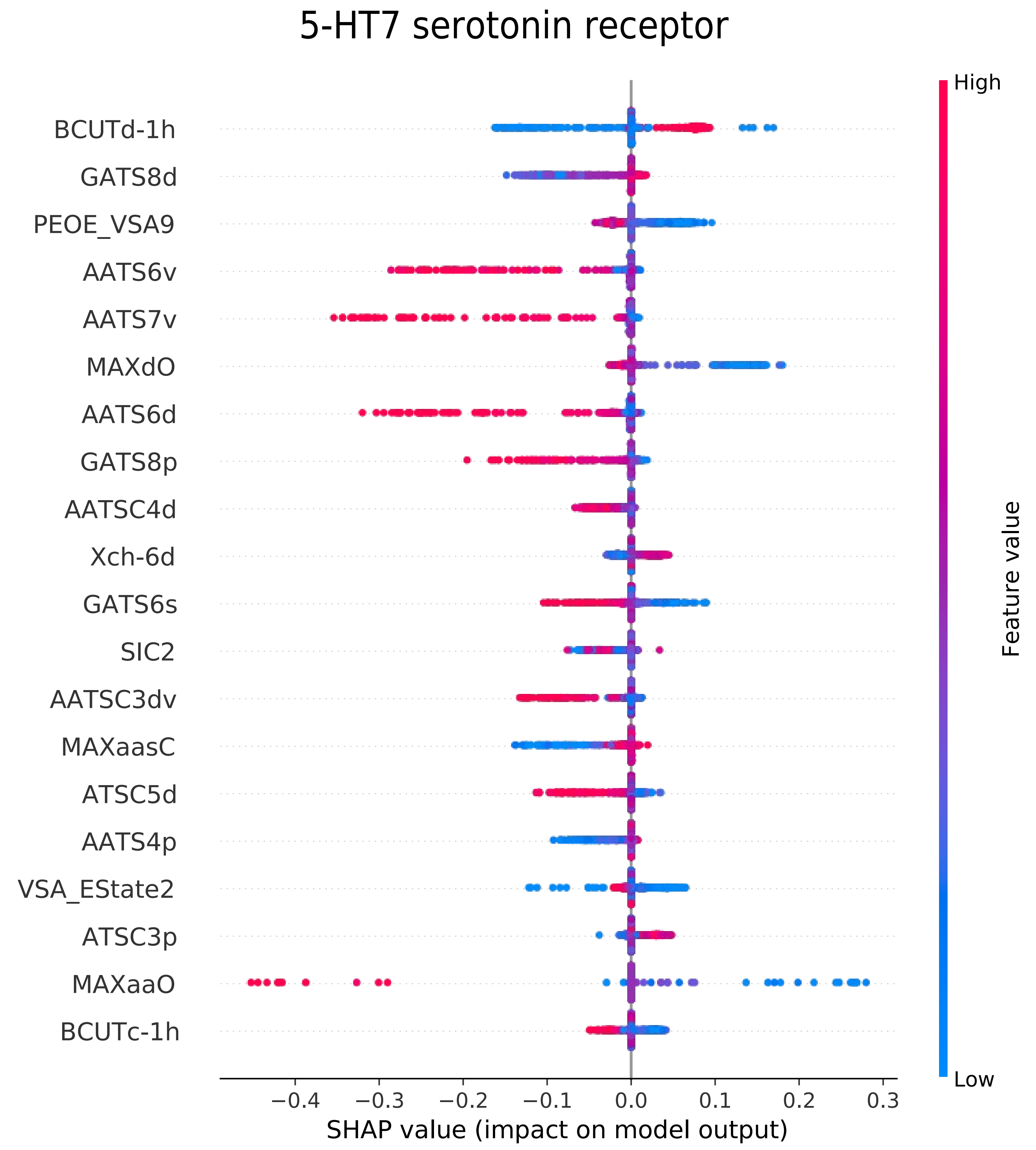

5-HT7 serotonin receptor
BCUTd-1h
GATS8d
PEOE_VSA9
AATS6v
AATS7v
MAXdO
AATS6d
GATS8p
AATSC4d
Xch-6d
GATS6s
SIC2
AATSC3dv
MAXaasC
ATSC5d
AATS4p
VSA_EState2
ATSC3p
MAXaaO
BCUTc-1h
−0.4
−0.3
−0.2
−0.1
0.0
0.1
0.2
0.3
SHAP value (impact on model output)
High
Low
Feature value

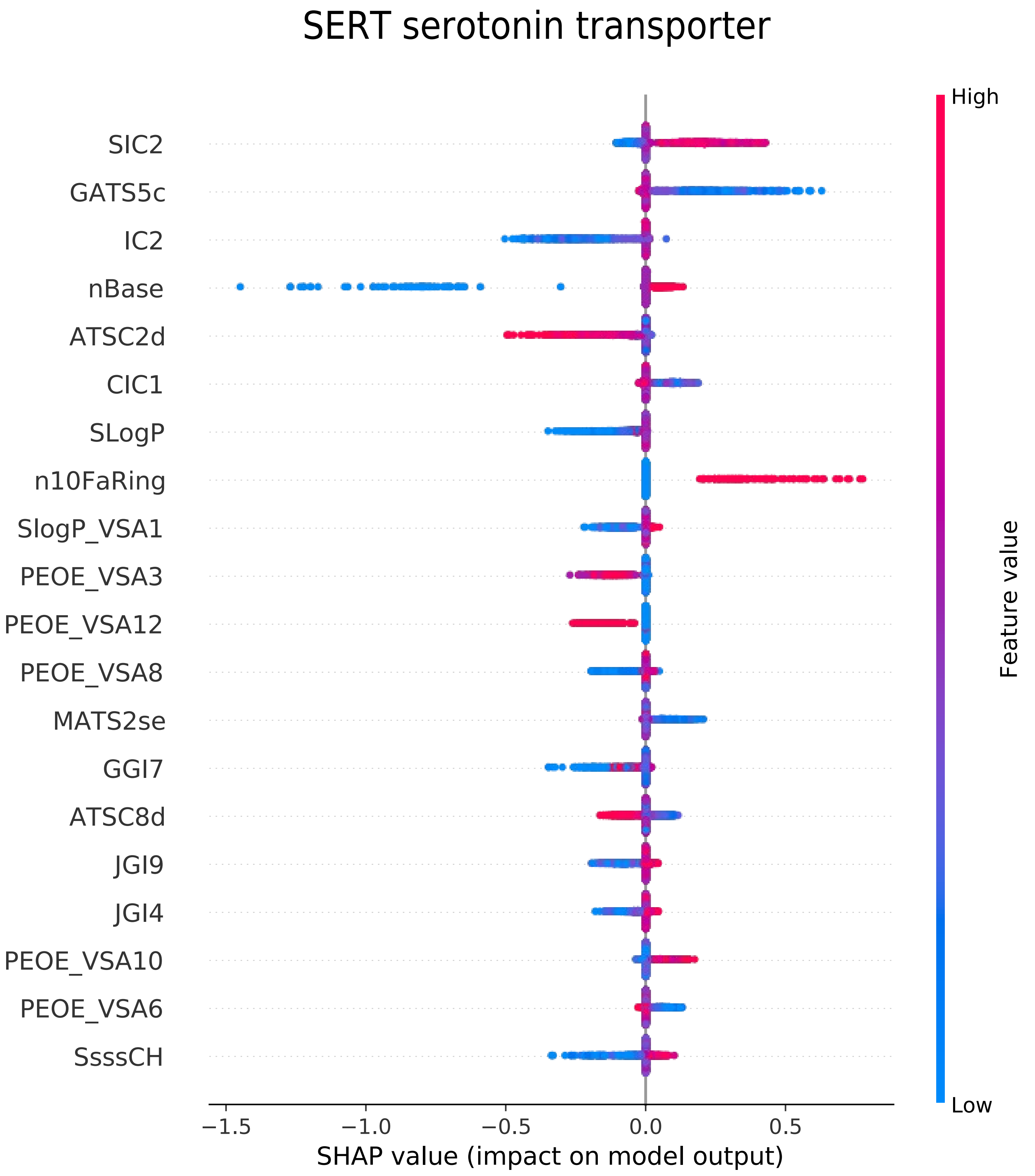
SERT serotonin transporter
SIC2
GATS5c
IC2
nBase
ATSC2d
CIC1
SLogP
n10FaRing
SlogP_VSA1
PEOE_VSA3
PEOE_VSA12
PEOE_VSA8
MATS2se
GGI7
ATSC8d
JGI9
JGI4
PEOE_VSA10
PEOE_VSA6
SsssCH
−1.5
−1.0
−0.5
0.0
0.5
SHAP value (impact on model output)
High
Low
Feature value

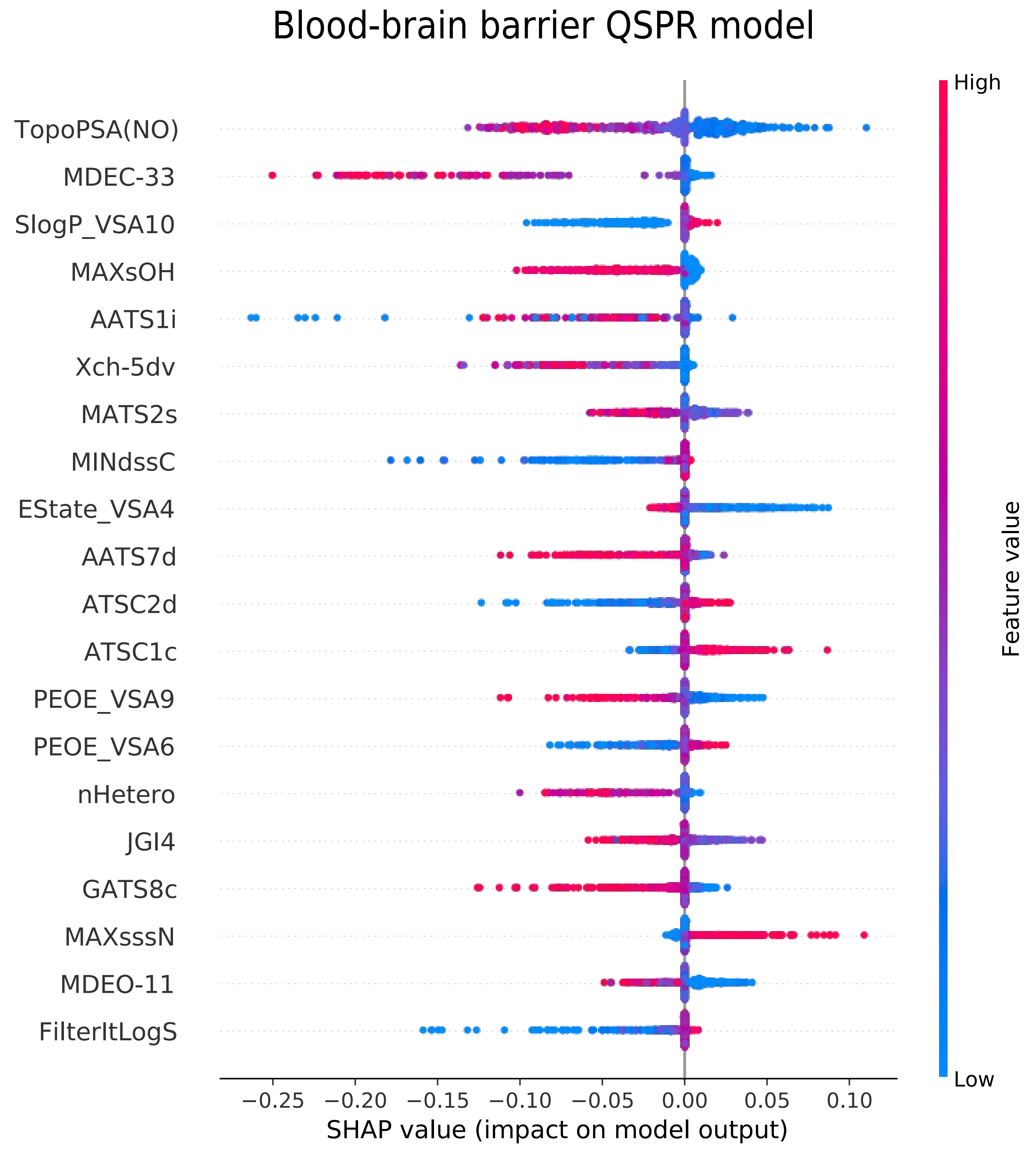
Blood-brain barrier QSPR model
TopoPSA(NO)
MDEC-33
SlogP_VSA10
MAXsOH
AATS1i
Xch-5dv
MATS2s
MINdssC
EState_VSA4
AATS7d
ATSC2d
ATSC1c
PEOE_VSA9
PEOE_VSA6
nHetero
JGI4
GATS8c
MAXsssN
MDEO-11
FilterItLogS
−0.25
−0.20
−0.15
−0.10
−0.05
0.00
0.05
0.10
SHAP value (impact on model output)
High
Low
Feature value
